# USMCA 2.0: a few improvements but far from a ‘healthy’ trade treaty

**DOI:** 10.1186/s12992-020-00565-4

**Published:** 2020-05-06

**Authors:** Ronald Labonté, Deborah Gleeson, Courtney L. McNamara

**Affiliations:** 1 School of Epidemiology and Public Health, Faculty of Medicine, University of Ottawa, 600 Peter Morand Crescent, Ottawa, Ontario K1G 5Z3 Canada; 2 School of Psychology and Public Health, La Trobe University, Melbourne, Australia; 3 Department of Sociology and Political Science, Norwegian University of Science and Technology, Trondheim, Norway

**Keywords:** USMCA, NAFTA 2.0, Protocol amendments, Trips+, Labour protection, Environmental protection

## Abstract

The USMCA (NAFTA 2.0), although signed over a year ago, went through several months of renegotiation of certain of its new rules that the Democrat-controlled US Congress wanted altered or strengthened. In December a ‘Protocol of Amendment’ was agreed upon and signed by the three Parties (the USA, Mexico, and Canada). A number of tough, new measures governing pharmaceuticals were revised or deleted, making it potentially easier for generic competition and lower drug costs in all three countries. Rules on protection of labour rights were also strengthened, lowering the threshold at which a complaint of unfair labour practices could be initiated. Procedures for investigating such a complaint or resolving a formal dispute were also improved. Similar procedural improvements were made on measures affecting environmental protection. These Protocol agreements are more health-positive than health-negative, and in the case of pharmaceuticals are of significant impact. Overall, however, these amendments are simply a political fine-tuning of the agreement. Concerns raised in our earlier health impact assessment of the USMCA, notably how the agreement’s regulatory reforms reduce public health policy flexibilities, remain. The agreement continues to subordinate known or potential health costs of many of its measures to dubious claims of aggregate economic gains. Moreover, these gains, if materialized, are likely to accrue to those atop the income/wealth hierarchies in all three nations.

## Background

When the USMCA (NAFTA 2.0) was signed (agreed upon) by the three member states (the USA, Mexico, and Canada) in late November 2018, it was already apparent that the Trump administration would have a challenge getting it ratified by a Democrat-controlled and trade-skeptical Congress. Key blocking issues for Congressional Democrats were the impact of the agreement on drug prices and labour and environment chapters that lacked sufficient enforceability. In the year that followed trade negotiators from all three countries took a second look at the specific Articles that looked like they would be deal busters, reaching agreement in December 2019 on a “Protocol of Amendment” to the USMCA [1]. The Protocol effectively changes several contentious measures that had previously been agreed upon.

In this brief update to our 2019 analysis of the USMCA’s health impacts [2], we focus on four key areas where Protocol changes move in a slightly more health-protective direction: pharmaceuticals, labour, environment, and dispute resolution. While these changes are welcome, our stance is that they do not fundamentally alter the health-negative implications of the agreement that we noted in our original assessment.

### Pharmaceuticals

Protocol amendments to the USMCA’s intellectual property rules for pharmaceuticals are extensive and are a significant positive development for public health, not just for the three nations involved but potentially also for other countries that negotiate with the US in future.

The USMCA as originally negotiated included 10 years of market exclusivity for biologic drugs, a highly contentious provision given rising concern about high drug prices. As widely anticipated, Protocol 3.E eliminates the USMCA’s biologic provisions, leaving it up to the three parties to determine for themselves the appropriate length of market exclusivity for these drugs, and leaving open the option of future domestic reforms. Also completely removed (Protocol 3.A) is the USMCA requirement that parties allow some form of secondary patenting for new uses of a known product, for new methods of a known product, or for new processes of using a known product, an ‘evergreening’ practice criticized for delaying generic competition. Similarly, the Protocol removes, scales back, or clarifies flexibilities associated with several other TRIPS-Plus^1^ elements in the original USMCA text which could have been expected to limit or delay generic competition.

The provisions that provide for data exclusivity (which prevents generic competitors from relying on patent holders’ test data for marketing approval purposes) are mitigated to some extent (Protocol 3.D.ii). The USMCA still provides for 5 years of data exclusivity for new pharmaceuticals, but the text that required parties to provide 3 years of additional data exclusivity for new clinical information is removed. The clause providing for 5 years of data exclusivity for combination products that include a previously approved drug, however, remains.

### Other protocol amendments to the USMCA

- Clarify the conditions and limitations that parties can apply to patent term adjustment (i.e. extension) for marketing approval delays, making it easier for parties to limit the length and number of extensions granted (Protocol 3.C).
- Clarify that the regulatory review exception has a broad scope, thereby smoothing the way for generic companies to have products ready for market launch as early as possible (Protocol 3.B).
- Remove the form of patent linkage most likely to limit generic competition for the USA and Canada (Protocol 3.F).
- Allow parties to reward those who successfully challenge patents and to provide procedures to promote transparency around patents and exclusivity periods (Protocol Article 3.F).

These Protocol amendments are progressive developments that are likely to encourage early launch of generics with their market-competitive potential to lower drug costs. They also represent a significant disruption to the previous ‘ratcheting up’ of IP provisions in successive US trade agreements [3].

Many other chapters in the USMCA have implications for access to medicines and other aspects of pharmaceutical policy, such as pricing and reimbursement, the assessment of safety and efficacy, and the ability of countries to support a viable generic medicines industry [4]. These parts of the text remain unaltered by the amendments, representing a lost opportunity for wider reforms to the US trade negotiating template.

#### Labour

Labour provisions within the USMCA were a major US congressional concern throughout the negotiations and will remain an issue in the implementation phase of the agreement. Concerns center on the enforceability of labour obligations and are also directly related to the outcome of the USA-Guatemala labour case under the trade agreement between the US and Central American states (CAFTA-DR). In this case, a dispute panel concluded that Guatemala had not breached CAFTA-DR labour provisions, as argued by the USA, even if the country had failed to effectively enforce its labour laws, as required by the agreement [5].

If, during the Protocol negotiations, the US Congress complained that the USMCA’s labour provisions lacked effective enforcement measures, Mexico was upset that the USA planned on sending five of its own labour inspectors to Mexico to ensure its compliance with the new rules, regarding this as an affront to sovereignty. Although all intergovernmental agreements imply some surrender of autonomous sovereignty, the US plan to rely on its own inspectors undermined the premise of the agreement's labour provision being the result of a joint determination. The Protocol largely assuages both concerns with the introduction of two Annexes (USA-Mexico and Canada-Mexico) that elaborates new procedural rules for a ‘Rapid Response Labor Mechanism’. This Mechanism creates and empowers an independent panel to undertake labour inspections. In the case of a suspected violation, inspection panelists will be drawn from among nine ‘experts in labor law and practice’, three each from both countries and three jointly agreed upon who are non-nationals of either country (Protocol 31.A.3). Both countries may have observers during verification of any claims of non-compliance (‘denial of rights’) by ‘a covered facility’ (a specific workplace), although only if both countries agree to do so (Protocol 31.A.7). The Mechanism also allows for the imposition of sanctions if the panel finds that a firm has not done enough to correct an issue (Protocol 31.A.4). As of mid-January 2020, however, discussions continue between the USA and Mexico over what much of this language means in practice [6, 7].

The Mechanism, however, does respond directly to concerns that the dispute panel in the USA-Guatemala case only included trade experts, and not labour ones [8]. Further, under the new Rapid Response Labor Mechanism, complaints can be filed directly against individual companies. This is in contrast to language in other trade agreements that specifies that violations can only be filed over governmental breaches of obligations. In this way, it seems that the bar for raising labour complaints has been lowered.

The USMCA requirement that a failure to comply with the chapter’s obligations applies only if it affects trade or investment between the countries remains. The responsibility to demonstrate such a failure, however, shifts from the complainant to the respondent country (Protocol 4.A.ii), with a dispute panel assuming the failure affects trade or investment unless the respondent country can demonstrate otherwise. This will make it easier for countries to initiate a complaint. In the case of protecting workers from violence, reference that any failure to do so must be of a ‘sustained and recurring course of action or inaction’ has been deleted (Protocol 4.E). Deleting this requirement lowers the threshold of what is needed to show that a violation with respect to violence, threats, and intimidation in a workplace is occurring. It also responds to another main concern from critics of the USA-Guatemala Panel Report: that there was not enough conceptual clarity around the language of a ‘manner affecting trade’ and ‘sustained and recurring course of action or inaction’.

The obligation to prohibit forced or compulsory labour has been strengthened by deleting the option that parties could do so ‘through measures it considers appropriate’ (Protocol 4.D). This appears to broaden the basis on which a complaint about forced/compulsory labour could be made. Finally, the lengthy and cumbersome process of consultations prior to initiating a formal dispute is eliminated by removal of a Free Trade Commission as part of the dispute settlement process (Protocol 7.A).

On balance, these Protocol amendments to the labour chapter could represent movement forward, broadening the bases to initiate a complaint and exchanging trade experts for labour experts in the make up of dispute panels. Given that the USA typically sets the template for labour provision language found in trade agreements, these changes are not insignificant, though their true force will ultimately depend on how the Protocol is implemented by the three states.

#### Environment

Several of the same changes to the labour chapter have been made in the environment chapter, including reversing the burden of proof that trade and investment was affected by a violation from complainant to respondent and eliminating the Free Trade Commission, thereby shortening the period before a dispute might be initiated. It also improves some language in some of the seven multilateral environment agreements (MEAs) referenced in the chapter, clarifying when failure to abide by an MEA is ‘in a manner affecting trade or investment between the Parties’, thereby increasing scope to make a complaint. If there is a conflict between any provision in the USMCA and any obligation under an MEA to which a country is party, the MEA obligations prevail (Protocol 1.A.1). Still absent from the list of MEAs, however, is the Kyoto Protocol.

Including reference to the Kyoto Protocol/climate change was an explicit ask from Canada in initial trade negotiations. It was also an ask of more than 110 House Democrats, who sent a letter to the president just a few months prior to the Protocol Amendment urging that the renegotiation “meaningfully address climate change” and “include binding climate standards and be paired with a decision for the United States to remain in the Paris Climate Agreement” [9].

#### Dispute resolution

More details are provided of how a roster of potential dispute panelists will be selected, specifying that each country can designate ten qualified individuals (hence thirty in total) for three-year terms (Protocol 7.C.1). Importantly, no country can block the re-appointment or selection of new roster members as terms expire. This was a concern, particularly for Canada, given that the Trump administration has refused to accept new nominations to the WTO’s appellate body, essentially eliminating its ability to enforce its dispute panel decisions. Trump’s WTO strategy reportedly intends to remove the USA ‘from the shackles of international trade rules’ in order to ‘use the power of its large market to force other countries to bend to its will’ [10]. The USMCA Protocol at least diminishes this outcome, insofar as formal dispute panels are concerned.

## Conclusion

Changes in the pharmaceutical rules and some improvements in the labour, environment, and dispute settlement chapters are welcome, however, they remain simply ‘tweaks’ that do not fundamentally alter the health-negative implications of the agreement we noted in our original assessment [2]. Fundamentally, the Protocol does not address concerns we raised about how the USMCA creates new, substantial, and enforceable measures affecting governments’ future regulatory policy space. It remains a commercial agreement that continues to subordinate known or potential health costs to dubious claims of aggregate economic gains which, in turn, and as we discussed in our previous article, are likely to accrue to those atop the income/wealth hierarchies in all three nations.

## Data Availability

Not applicable.

## Footnotes

1 TRIPS-Plus refers to measures that go beyond those in the World Trade Organization’s Agreement on Trade-Related Intellectual Property Rights (TRIPS).
